# Depression and Anxiety in patients undergoing Percutaneous Coronary Intervention for Acute Coronary Syndrome

**DOI:** 10.12669/pjms.36.5.1749

**Published:** 2020

**Authors:** Syed Fayaz Mujtaba, Jawaid Akbar Sial, Musa Karim

**Affiliations:** 1 Syed Fayaz Mujtaba, National Institute of Cardiovascular Diseases, Karachi, Pakistan; 2 Jawaid Akbar Sial, National Institute of Cardiovascular Diseases, Karachi, Pakistan; 3 Musa Karim, National Institute of Cardiovascular Diseases, Karachi, Pakistan

**Keywords:** Acute Coronary Syndrome (ACS), Anxiety, Depression, Percutaneous Coronary Intervention (PCI)

## Abstract

**Background and Objective:**

Depression and anxiety are very common in patients with cardiac diseases. Percutaneous coronary intervention (PCI) has not only decreased mortality but angina, heart failure and recurrent hospitalization all are improved. Therefore, anxiety and depression associated with fibrinolytic therapy in acute coronary syndrome (ACS) are expected to be decreased in the patient undergoing PCI. Therefore, the aim of this study was to determine prevalence of depression and anxiety in patients undergoing percutaneous coronary intervention for acute coronary syndrome.

**Methods:**

This study was conducted at Larkana Satellite Center of National Institute of Cardiovascular Diseases (NICVD), Pakistan from August 2018 to December 2018. Patients who underwent cardiac intervention within one month were enrolled in this study. Patients were interviewed regarding their basic demographics, indication for intervention and procedure details. Symptoms of anxiety were assessed using the translation of inventory of SAS (Zung’s Self-Rating Anxiety Scale). Patients were interviewed for depression by using Becks depression inventory (BDI).

**Results:**

A total of 153 patients were included in this study out of which 118 (77.1%) were males and 35 (22.5%) were females. All were married except one. Diabetes mellitus (DM) was present in 61 (39.9%), hypertension (HTN) in 69 (45.15%), obesity in 15 (9.8%), and 40 (26.1%) were smokers. Depression was present in 16 (10.5%) patients and anxiety was present in 12 (7.5%) of patients. On analysis of the association of various factor with depression; non-diabetics, housewives, laborers and uneducated were found to be more depressed. While those who smoke or earn more than 50 thousand were found less likely to be depressed.

**Conclusion:**

Both depression and anxiety were present in only 10.5% and 7.5% of the patients after percutaneous coronary intervention for acute coronary syndrome.

## INTRODUCTION

Depression and anxiety are very common in heart patients. Chronic heart conditions like a left ventricular failure^1^,^2^ and pulmonary hypertension^3^ have a very high frequency of anxiety and depression. Acute coronary syndrome (ACS) is a different disease process in a way that most of the suffering patients never had such severe pain before. The pain of ACS, especially myocardial infarction has been labeled as one of the severest of its kind, reminding doomsday.^4^ Therefore, ACS has a profound psychological effect.^5^

Many studies have shown an association of ACS with depression and anxiety. It has been found 46.7% in one study,^6^ 43.5% in Brazil by using the becks depression inventory (BDI),^7^ and 20% in the United States of America (USA).^8^ In the era of percutaneous coronary intervention, things have changed. Not only mortality is reduced but pain, failure and hospitalization all are improved. Therefore, anxiety and depression related to fibrinolytic are expected to be decreased in the patient undergoing PCI.

Assessing depression and anxiety is not an easy job. In developing countries where healthcare facilities are not easily available, priority is always the provision of specific treatment of disease; hence psychological aspects of diseases are often neglected. There are many tools for the assessment of depression and anxiety with reasonable validity. Assessment of depression and anxiety in a rural setup like ours, where most of the patients are not well education is difficult. Therefore, we used the translated version of validated tools.

## METHODS

Patients who underwent cardiac intervention within last one month were enrolled in this study. Patients were enrolled from the outpatient department (OPD) of a rural satellite center (Larkana), of National Institute of Cardiovascular Diseases (NICVD), Pakistan from August 2018 to December 2018. Patients who had a prior history of the psychological problem (depression, anxiety, suicidal tendency) were excluded. Patients with other chronic illness like arthritis, chronic renal failure (CRF). malignancy and debilitating cerebral stroke were also excluded. After taking informed consent, patients were interviewed regarding their basic demographics, indication for intervention, procedure details. Patients were also asked about education level and their economic status. Patient who could read newspaper of English or any local language was labelled as educated. Patients were divided in three financial classes according monthly income; such as less than 10 thousands ($70), 10 thousands to 50 thousands ($70-350) and above 50 thousand ($350) Ethical Approval (SMBBMU/OFF ERC/109, Dated: 02-03-2019).

Symptoms of anxiety were assessed using the translation of inventory of SAS (Zung’s Self-Rating Anxiety Scale). It contains 20 items. The respondents were asked a four-point scale to assess all statements (from never / rarely, to very often / all the time). Patients were categorized in anxiety groups based on anxiety score, patients with a score of 0-8 were categorized as minimal, 9-16 as mild, 17 to 24 as moderate, 25-32 as high, and 33 and above as extreme anxiety. Patients having moderate or more anxiety (>16) were labeled as having anxiety.

Similarly, patients were interviewed for depression by using Becks depression inventory (BDI). Patients were categorized into four groups based on BDI score, patients with DBI score of 0-13 were categorized as minimal, 14-19 as mild, 20-28 as moderate, and 29 or more as severe depression. Patients having mild depression or more (DBI score >13) were labeled as having depression.

Collected data were entered and analyzed using IBM SPSS Statistics for Windows, Version 21.0. (IBM Corp., Armonk, NY, US). The hypothesis of normality of distribution of SAS and DBI score were assessed by applying Shapiro-Wilk normality test. The Mann-Whitney U test or Kruskal-Wallis for the score comparison by different baseline characteristics and the Chi-Square test or Fisher’s exact test for the categorical comparison by different baseline characteristics were applied.

## RESULTS

A total of 153 patients were enrolled in this study. Mean age was 52.15±10.59 years. Males were 118 (77.1%) and females were 35 (22.9%). Most of the patients, 99.3% (152), were married, and hypertensive and diabetic patients were 45.1% (69) and 39.9% (61) respectively. Demographic features of the patients are presented in Table-I

**Table-I:** Baseline characteristics of patients.

Characteristics	Total (n = 153)
Age (years)	52.15 ± 10.56
Male	77.1% (118)
Married	99.3% (152)
Employed	60.1% (92)
Educated	43.8% (67)
***Risk factors***	
Diabetes	39.9% (61)
Hypertension	45.1% (69)
Obesity	9.8% (15)
Smoking	26.1% (40)
***Financial status***	
Less than 10 thousand ($70)	48.4% (74)
10 to 50 thousand ($70-350)	46.4% (71)
Above 50 thousand ($350)	5.2% (8)

The mean anxiety score was 5.88±6.18 with the majority of the patients, 75.2% (115), fall under the minimal anxiety category, while, 17.0% (26) had mild anxiety, 6.5% (10) had moderate anxiety, and 1.3% (two) had high anxiety. Overall moderate to high anxiety was observed in 7.8% (12) patients.

**Fig.1:**
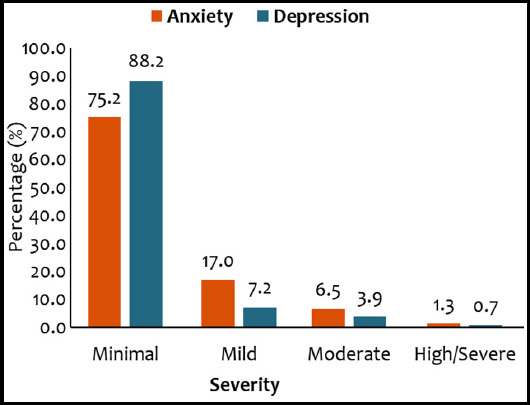
Distribution of patients by severity of anxiety and depression.

The mean depression (BDI) score was 6.20±6.42 with the majority of the patients, 88.2% (135), fall under the minimal depression category, while, 7.2% (11) had mild depression, 3.9% (six) had moderate depression, and 0.7% (one) had severe depression. Overall mild to severe depression was observed in 11.8% (18) patients. Severity distribution of patients by anxiety and depression are presented in Fig.1.

The mean anxiety score was significantly higher among female patients. However, no differences were observed in the level of anxiety of the patients by their demographic characteristics. Assessment of anxiety score by baseline characteristics of the study sample is presented in Table-II.

**Table-II:** Assessment of Anxiety score by baseline characteristics of the study sample.

Characteristics	Base (N)	Anxiety Score		Anxiety	

		Mean ± SD	p-value	Frequency (%)	p-value
***Gender***					
Male	118	5.48 ± 6.42	0.010*	8.5% (10)	0.594
Female	35	7.23 ± 5.1		5.7% (2)	
Married	152	5.91 ± 6.18	0.471	7.9% (12)	0.770
Unmarried	1	1 ± 0		0% (0)	
***Diabetes***					
Yes	61	6.03 ± 6.16	0.506	8.2% (5)	0.895
No	92	5.78 ± 6.21		7.6% (7)	
***Hypertension***					
Yes	69	6.64 ± 6.64	0.270	10.1% (7)	0.337
No	84	5.26 ± 5.73		6% (5)	
***Obesity***					
Yes	15	7.27 ± 5.08	0.131	6.7% (1)	0.858
No	138	5.73 ± 6.28		8% (11)	
***Smoking***					
Yes	40	5 ± 6.01	0.118	7.5% (3)	0.925
No	113	6.19 ± 6.23		8% (9)	
***Employment status***					
Employed	67	5.24 ± 6.11	0.123	9% (6)	0.087
Unemployed	86	6.38 ± 6.21		7% (6)	
***Education status***					
Educated	92	5.61 ± 6.55	0.170	10.9% (10)	0.652
Uneducated	61	6.3 ± 5.58		3.3% (2)	
***Financial status***					
Less than 10 thousand ($70)	74	5.89 ± 6.63	0.134	8.1% (6)	0.696
10 to 50 thousand ($70-350)	71	6.28 ± 5.89		8.5% (6)	
Above 50 thousand ($350)	8	2.25 ± 2.43		0% (0)	

No significant differences were observed in the mean depression (BDI) score by the demographic characteristics of the patients. However, depression was observed in a significantly higher number of uneducated and non-diabetic patients as compared to educated and diabetic patients. Assessment of depression score by baseline characteristics of the study sample is presented in Table-III.

**Table-III:** Assessment of depression score by baseline characteristics of the study sample.

Characteristics	Base (N)	Depression Score		Depression	

		Mean ± SD	p-value	Frequency (%)	p-value
***Gender***					
Male	118	5.86 ± 6.43	0.153	11% (13)	0.598
Female	35	7.37 ± 6.32		14.3% (5)	
Married	152	6.23 ± 6.43	0.654	11.8% (18)	0.714
Unmarried	1	2 ± 0		0% (0)	
***Diabetes***					
Yes	61	4.93 ± 5.19	0.083	4.9% (3)	0.032
No	92	7.04 ± 7.02		16.3% (15)	
***Hypertension***					
Yes	69	5.8 ± 6.05	0.632	8.7% (6)	0.286
No	84	6.54 ± 6.73		14.3% (12)	
***Obesity***					
Yes	15	4.07 ± 2.94	0.421	0% (0)	0.136
No	138	6.43 ± 6.65		13% (18)	
***Smoking***					
Yes	40	4.6 ± 5.2	0.069	7.5% (3)	0.330
No	113	6.77 ± 6.73		13.3% (15)	
***Employment status***					
Employed	67	4.54 ± 3.96	0.894	3% (2)	0.673
Unemployed	86	7.5 ± 7.59		18.6% (16)	
***Education status***					
Educated	92	5.97 ± 5.99	0.066	10.9% (10)	0.003*
Uneducated	61	6.56 ± 7.05		13.1% (8)	
***Financial status***					
Less than 10 thousand ($70)	74	6.82 ± 6.91	0.590	13.5% (10)	0.790
10 to 50 thousands ($70-350)	71	5.72 ± 5.93		9.9% (7)	
Above 50 thousands ($350)	8	4.75 ± 6.02		12.5% (1)	

## DISCUSSION

Depression is a mental health disorder wherein low mood and low energy can affect a person’s thoughts, feelings, behavior, and sense of well-being.^9^ Many studies have showed that depression and anxiety are common in patients suffering from medical illness. Depression is a key element in patients with diabetes and hypertension; two leading causes of coronary artery disease (CAD).^10^

We found less than 10% of patients with ACS are having depression and anxiety. Our results are lower than other studies conducted in Pakistan.^11^,^12^ One recent study indicates that depression is common in rural areas of Pakistan.^13^ Reasons may be twofold. Most of the time patients coming into cardiac clinic are not asked about depression and anxiety symptoms. Therefore, patients are not forthcoming with their response. While asking questions about depression and anxiety we had to repeat or rephrase over two to three times in some cases to make them speak. Other studies too have shown that identification of depression remains undiagnosed and untreated in many patients.^14^ Secondly we conducted study in outpatient setting after the patient is discharged. While other studies mentioned are in hospital studies. Therefore, frequency of anxiety and depression is lower in our study.

As compared to western literature, our study showed decreased frequency of anxiety and depression in ACS patients. First, our patients are not usually asked about the psychological aspects of diseases. Therefore, they were finding difficulty in understanding questions. Secondly reply to certain questions is considered as taboo or sign of cowardice in our society. Especially, in rural society it is considered cowardice to complain about feeling down or want to cry. Thirdly, in rural areas, the family system is very strong. Especially in circumstances of illness even far way relatives come for help and assuage. Patients don’t have to worry much about the financial state, especially at the start of the illness. A heart attack is considered major illness and friends and relatives provide strong moral support during the initial phase^9^ Other studies also show that a family and social system plays an important role in decreasing depression.^15^,^16^

On analysis of the association of risk factors with the frequency of depression, one important contradiction was identified. We found that depression was less common in diabetics. While many studies show that chronic conditions like diabetes are associated with increased depression.^17^-^19^ In a study of 133 diabetic patients, 38% were found depressed, and depression rate was significantly higher among female patients.^20^ Himelhoch et al. illustrated that emergency room visits are two to three times more common among patients with diabetes and hypertension who have depression as opposed to chronically ill patients without depression.^21^

Reason of this seemingly surprising finding can be that non-diabetics were almost free of any medical illness before this event. While diabetics do have multiple diseases or co-morbid. Diabetics are often on chronic medication. Therefore, they were facing some medical problem even before this event. But for non-diabetics ACS might have been first medical presentation and admission, therefore they were unable to cope up with the present illness. The same phenomenon is demonstrated by another study that patient presenting with ACS and history of past myocardial infarction are less likely to be depressed than those who present with ACS without previous history of heart attack.^22^

We found female had comparatively more depression than the male counterpart. Similar results have also been shown in other studies.^23^ Our study showed that less educated were found more depressed. Same has been shown in other studies across the world.^24^-^28^ Like other studies, our study too showed that patients with low socioeconomic status were having more depression than those financially stable.^29^,^30^

This may be because of the cost of cardiac medication and stage PCI procedure which a post PCI patient has to arrange. In our study most of the patients belonged to the poor economic status. Post ACS patients are advised multiple medicines. These patients have never been on chronic medication before. ACS and subsequent procedure put them an extra burden of cost. This stress may expose them to depression and anxiety.

Our study was unique in many ways. First, we used language translation of both assessment tools. Secondly, patients were interviewed rather than filling the form themselves, thirdly majority of our population belonged to the rural area having less number of educated patients. Fourth our population mostly belonged to the poor class who received free medical care and interventions. Fifth we assessed the latest method of ACS management i.e. PCI. Therefore, we expected different results than those most widely published earlier.

### Limitations of the study.

We used Sindhi translation of Becks depression inventory (BDI) and SAS (Zung’s Self-Rating Anxiety Scale) scoring system. These translations are not validated yet in large studies. Secondly due to the fact that majority of our patients were not able to read we had to ask the question verbally. Thirdly our study setting was an outpatient clinic and population based study may be conducted to determine frequency more accurately.

## CONCLUSIONS

Both depression and anxiety were present in only 10.5% and 7.5% of the patients after percutaneous coronary intervention for acute coronary syndrome. Both depression and anxiety were found less common in rural population as compared to the urban-based studies. However, still, there are subgroups like females, less educated and patients belonging to low socioeconomic state, where prevalence is significant.

### Authors’ Contribution

**SFM:** Conceived, designed, did data collection a manuscript writing. Responsible and accountable for the accuracy and integrity of the work.

**JAS & MK:** Did statistical analysis & editing of manuscript.
